# Discovery of dual-activity small-molecule ligands of *Pseudomonas aeruginosa* LpxA and LpxD using SPR and X-ray crystallography

**DOI:** 10.1038/s41598-019-51844-z

**Published:** 2019-10-29

**Authors:** Kyle G. Kroeck, Michael D. Sacco, Emmanuel W. Smith, Xiujun Zhang, Daniel Shoun, Afroza Akhtar, Sophie E. Darch, Frederick Cohen, Logan D. Andrews, John E. Knox, Yu Chen

**Affiliations:** 1 Department of Molecular Medicine, University of South Florida, 12901 Bruce B. Downs Boulevard, Tampa, Florida 33612 United States; 2 Former employees of ACHAOGEN Inc., 1 Tower Place, Suite 400, South San Francisco, California 94080 United States

**Keywords:** Biochemistry, Chemical biology, Drug discovery, Structural biology

## Abstract

The lipid A biosynthesis pathway is essential in *Pseudomonas aeruginosa*. LpxA and LpxD are the first and third enzymes in this pathway respectively, and are regarded as promising antibiotic targets. The unique structural similarities between these two enzymes make them suitable targets for dual-binding inhibitors, a characteristic that would decrease the likelihood of mutational resistance and increase cell-based activity. We report the discovery of multiple small molecule ligands that bind to *P. aeruginosa* LpxA and LpxD, including dual-binding ligands. Binding poses were determined for select compounds by X-ray crystallography. The new structures reveal a previously uncharacterized magnesium ion residing at the core of the LpxD trimer. In addition, ligand binding in the LpxD active site resulted in conformational changes in the distal C-terminal helix-bundle, which forms extensive contacts with acyl carrier protein (ACP) during catalysis. These ligand-dependent conformational changes suggest a potential allosteric influence of reaction intermediates on ACP binding, and vice versa. Taken together, the novel small molecule ligands and their crystal structures provide new chemical scaffolds for ligand discovery targeting lipid A biosynthesis, while revealing structural features of interest for future investigation of LpxD function.

## Introduction

Antibiotic resistance is a worldwide threat that challenges our ability to successfully treat bacterial infection, exhausting health care resources and worsening patient prognosis. Infections caused by antibiotic-resistant Gram-negative bacteria (GNB) are particularly intractable due to the presence of the outer membrane that protects the bacterial cell from harsh environments and antibiotics^1^. The outer leaflet of this membrane consists primarily of lipopolysaccharide (LPS)^2^. Although LPS appears dispensable in some pathogens (e.g. *Acinetobacter baumannii, Neisseria meningitidis*), it is essential in many clinically important GNB such as *Pseudomonas aeruginosa*, a common and potentially life-threatening nosocomial pathogen that is naturally resistant to many antibiotics^1–6^. There are currently no clinical antibiotics targeting LPS synthesis, but research has shown that compounds inhibiting this biochemical pathway provide an excellent opportunity for the development of new antibiotics with a novel mechanism of action^7,8^. LPS is made of three components: (1) a linear chain of repeating saccharide units known as the O-antigen, (2) an oligosaccharide core domain, and (3) lipid A, a glucosamine disaccharide that is connected to multiple fatty acid chains of various lengths^2,6^. Lipid A has two interesting properties that underscore its importance to GNB. The first is that lipid A is the minimal component of LPS required for cellular viability in most GNB^2,3,9^. Secondly, lipid A is the primary antigenic determinant of LPS and is the offending chemical species that triggers septic shock^2,10,11^.

**Figure 1:**
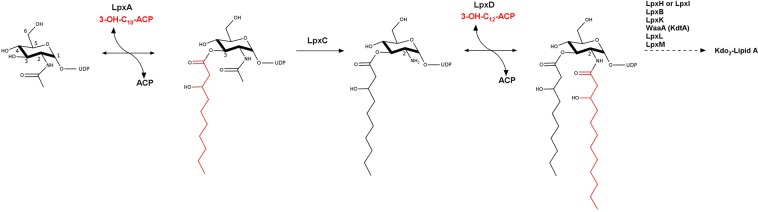
Lipid A biosynthesis pathway. In *Pseudomonas aeruginosa* LpxA catalyzes the first step in the lipid A biosynthetic pathway (Raetz pathway) by mediating the reversible transfer of R-3-hydroxydecanoate from the acyl carrier protein (ACP) onto the 3-OH position of UDP-GlcNAc forming an ester bond. LpxD catalyzes the third step in the Raetz pathway, the reversible transfer of a R-3-hydroxydodecanoate from ACP onto the 2-NH_2_ of the UDP-3-*O*-(3-hydroxydecanoyl) glucosamine through the formation of an amide bond.

The lipid A biosynthetic pathway, also known as the Raetz pathway, is highly conserved amongst all GNB. LpxA, LpxC and LpxD make up the first three enzymes in the Raetz pathway (Fig. 1)^2^. In *P. aeruginosa*, LpxA catalyzes the first step, transferring an R-3-hydroxydecanoic fatty acid to the uridine diphosphate N-acetylglucosamine (UDP-GlcNAc) substrate using acyl carrier protein (ACP) as the R-3-hydroxydeconoate donor (3-OH-C_10_-ACP). In the second step, LpxC catalyzes the zinc-dependent irreversible deacetylation of the LpxA product, producing UDP-3-*O*-(3-hydroxydecanoyl) glucosamine and committing the molecule to this pathway. LpxD is responsible for the third step, in which an R-3-hydroxydodecanoate is transferred to the 2’ amine of UDP-3-*O*-(3-hydroxydecanoyl) again using an ACP donor^12^. Six additional enzymatic steps are required before the completed lipid A can be incorporated into the LPS molecule through attachment to the core component (Fig. 1)^2,13–15^.

While LpxC is not homologous to LpxA and LpxD, the latter two enzymes share several unique structural features, consistent with their functional similarities in catalyzing the transfer of a 10 or 12 carbon chain fatty acid from ACP to UDP-GlcNAc through a concerted acid-base mechanism^16,17^. Both proteins form biological homotrimers that contain a left-handed helical fold comprised of multiple parallel β-sheets^14,16–24^. Although *P. aeruginosa* LpxA and LpxD only share 27% sequence identity, they exhibit highly conserved protein backbone and side chain structural features, particularly at the junctions of adjacent β-helix monomers that form the acyl chain binding pocket.

LpxC has been extensively targeted in antibiotic discovery and many potent small-molecule inhibitors with bactericidal properties have been developed^25^. In contrast, inhibitor discovery against LpxA and LpxD has remained largely unexplored with no published small molecule inhibitors (only peptide inhibitors) having ever been identified^26–29^. The unique, shared structural similarities also make LpxA and LpxD amenable to dual-targeting inhibitors, which offer the advantage of increased potency and reduced likelihood of resistance formation^30^. The concept of dual targeting the early steps of the lipid A biosynthetic pathway has previously been demonstrated with a peptide molecule RJPXD33, found to inhibit both LpxA and LpxD when expressed in *E. coli*^27^. Herein we report the discovery of several small molecules that bind to both *P. aeruginosa* LpxA and LpxD with μM affinity, identified using a targeted structure-based methodology that utilizes molecular docking, surface plasmon resonance (SPR) bioanalysis, and high-resolution X-ray crystallography. The structural analysis has provided valuable insights regarding inhibitor binding hot spots of LpxA and LpxD, and allosteric effects induced by ligand binding in the LpxD active site.

## Results

### LpxD crystallization

The crystal structures of both *P. aeruginosa* LpxA and LpxD have been determined previously^18,24^. In the published *P. aeruginosa* LpxD structure however, the thrombin protease recognition sequence of the N-terminal His-tag linker is located in the active site, occupying the uracil binding pocket. This obstructs the diffusion of small molecules into the active site, preventing the use of this construct in both functional and structural studies of ligand binding. After failed attempts to crystallize untagged LpxD following protease cleavage, we tested several variations of this LpxD construct by first replacing the thrombin protease cleavage site with a TEV protease recognition sequence, and then excising the first two residues of the LpxD sequence, which are located immediately after the N-terminal 18-AA hexahistidine tag and protease site. The resulting construct led to protein crystals similar to the published one, with each asymmetric unit of the H3 space group containing one monomer that forms a biologically relevant homotrimer through 3-fold crystallographic symmetry operation^18^. Importantly, in the apo structure of the new LpxD construct determined at 1.55 Å resolution, the density of the His-tag linker is no longer observed in the active site. In fact, whereas these residues are ordered in the previous structure, this entire region of the His-tag and protease site is disordered in the current structure. However, our failure to crystallize untagged LpxD suggests the His-tag may still contribute to the stability of the crystal-packing interface, despite the lack of an ordered conformation.

**Figure 2:**
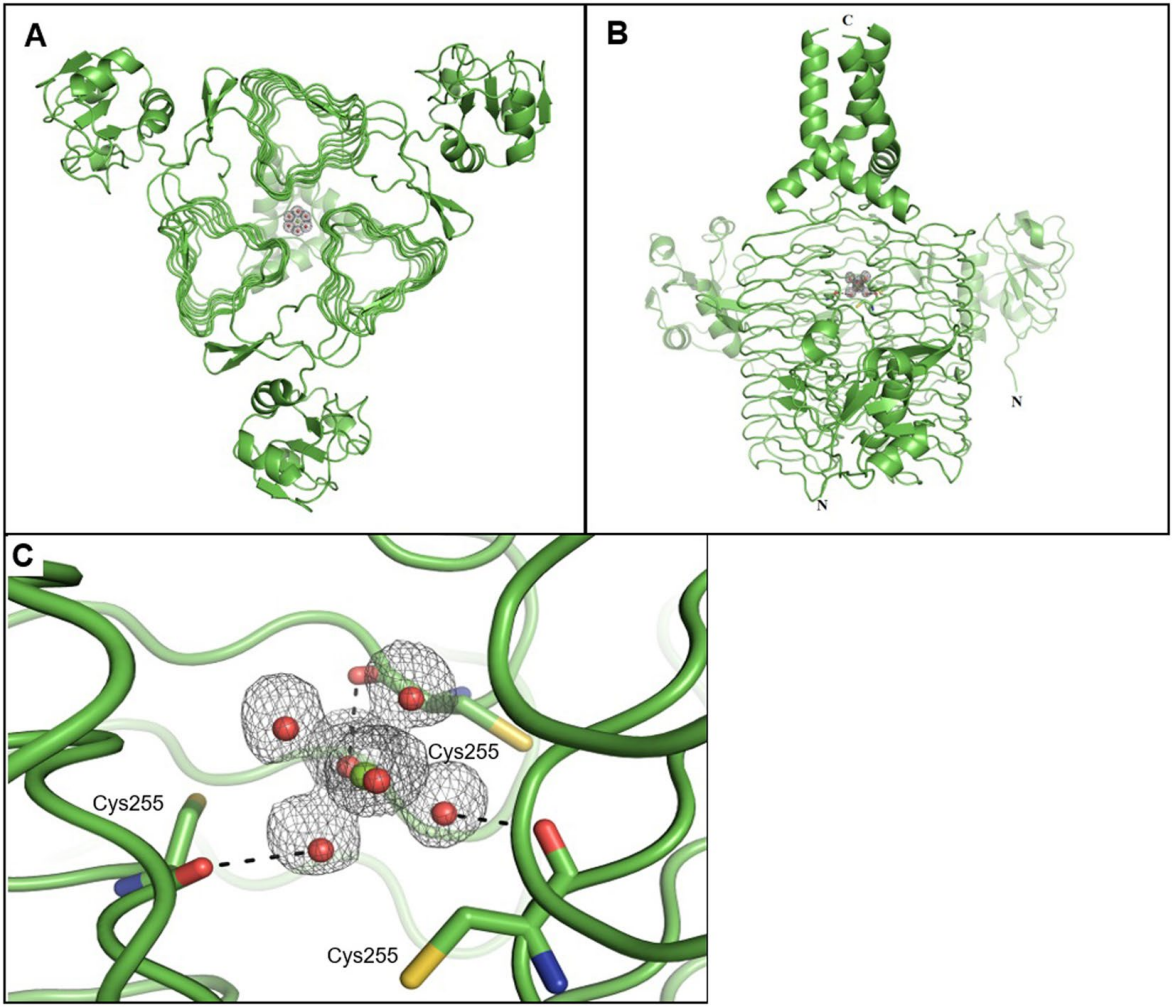
Magnesium ion and coordinated waters at the core of LpxD trimer. The 2Fo-Fc electron density map, determined at 1.55 Å resolution, is shown at 1.5 σ. **(A)** Overhead view of the LpxD trimer (green) with well-defined electron densities of a magnesium ion (green sphere) coordinating with six water molecules (red spheres) at the core of the trimer. **(B)** Side view of LpxD with the C-terminal helix bundle shown at the top. **(C)** Zoomed in side view of LpxD and the magnesium ion. Hydrogen bonds are indicated by black dashed lines.

An interesting observation in our new LpxD structure is a well-defined magnesium ion in the core of the trimer (Fig. 2). This magnesium ion, likely from the crystallization buffer, coordinates six water molecules and appears to be critical to the stability of the crystal, as removing magnesium from the crystallization buffer or chelating the magnesium with EDTA eradicates X-ray diffraction by the crystal. A similar, but slightly weaker density corresponding to this magnesium ion was observed in the previously published structure crystallized in the absence of magnesium in the crystallization buffer (PDB ID: 3PMO, 1.30 Å resolution)^18^. Although it was modeled as water, the surrounding density peaks suggest that this density in the previous structure may be a low-occupancy divalent metal ion coordinating six water molecules as observed in the structure reported here.

### Structure-based ligand discovery

In the absence of a functional high-throughput assay and any previously discovered small molecule ligands against *P. aeruginosa* LpxA/D, virtual screening of the ZINC small molecule database was performed using DOCK 3.5 to identify potential compounds that would bind to the LpxA active site^31,32^. Specifically, the acyl chain binding pocket was targeted because of similar binding surfaces shared between LpxA and LpxD (such as the backbone groups of Gln157 and Gly151 in LpxA, corresponding to those of Gly238 and Gly272 of LpxD, among other shared structural features). Top scoring compounds were visually inspected. 25 of these compounds were selected and experimentally assessed against LpxA and LpxD with SPR bioanalysis. Five of these compounds (20%) demonstrated approximately micromolar affinity for LpxA, and 2 of these 5 retained similar affinity for LpxD (compounds **1**–**5**, Table 1, Supplementary Figs S1 and S2). It should be mentioned that the data quality for some compounds (i.e., **1** and **2**) was lower than others (Supplemental Fig. S1). We included these compounds as positive results because their binding kinetics were distinct from non-binders, and because, as described below, crystal structures suggested that they were likely binders. To further exclude the possibility of non-specific protein binding caused by small molecule aggregation^33,34^, we analyzed compounds **1** and **2** against two unrelated enzymes, CTX-M-14 and KPC-2 Class A β-lactamase^35,36^, in a nitrocefin-hydrolysis assay using the SPR buffer. The compounds showed no inhibition of CTX-M-14 or KPC-2 even when tested at 3 mM concentrations, suggesting that the binding to LpxA and LpxD was specific.

**Table 1:** Binding affinity (Kd) determined by surface plasmon resonance assay.

Compound	Structure	LpxA (µM)	Χ^2^ (LpxA)	LpxD (µM)	Χ^2^ (LpxD)
1		NA	NA	NA	NA
2		19.5	0.2196	36.7	0.2282
3		16.7	4.124	NB	NB
4		13.6	0.3219	NB	NB
5		2.1	0.4179	NB	NB

^a^NA = Kd could not be determined via fitting due to poor data quality; NB = No Binding.

^b^Χ^2^: The average deviation of the experimental data from the fitted curve, where lower numbers indicate a better fit. Each compound concentration was tested in triplicates and all data were fitted onto one dose-response curve.

### Complex crystal structures with small molecule ligands

**Figure 3:**
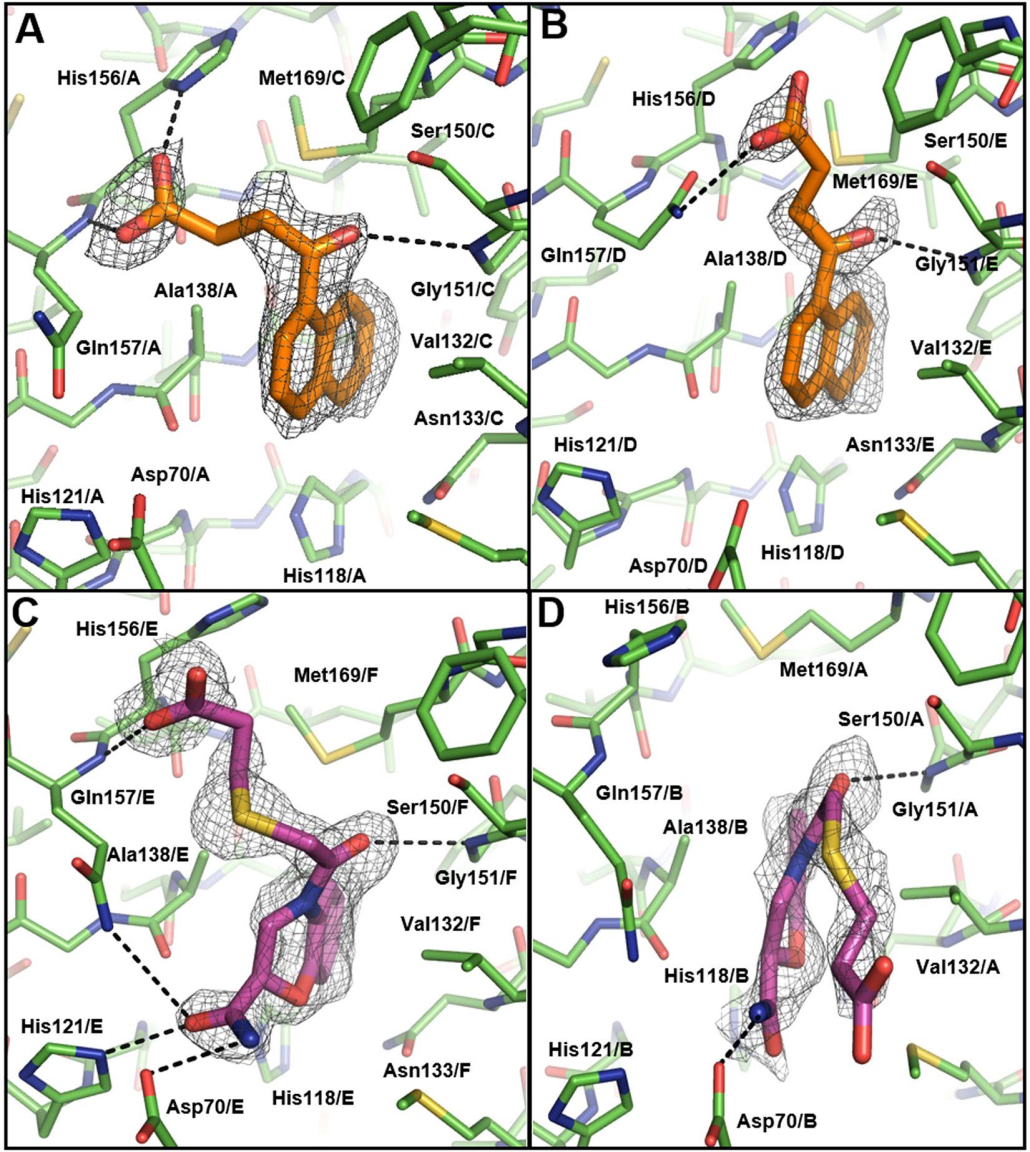
LpxA complex structures with novel inhibitors. Compounds **1** (orange) and **2** (purple) bind to both monomers at the dimer interface of LpxA (green). The chain IDs (A/B/C/D/E/F) indicate different monomers constituting the active sites. Potential hydrogen bonds are shown as dashed lines. The unbiased Fo – Fc map are contoured at 2.0 σ. Two unique binding modes are observed for each compound in different LpxA active sites of the same trimer. **(A)** Pose 1 of compound **1**. **(B)** Pose 2 of compound **1**. **(C)** Pose 1 of compound **2**. **(D)** Pose 2 of compound **2**.

**Figure 4:**
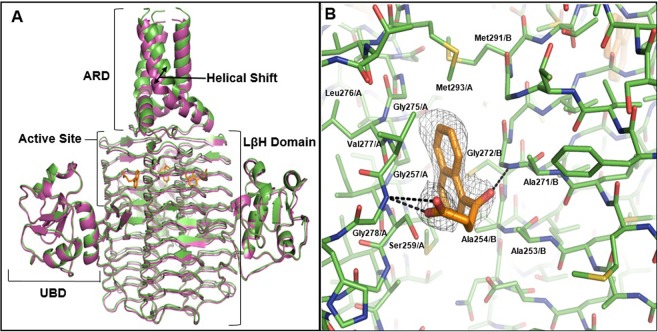
LpxD complex structure with compound 1. The compound is colored in orange. Potential hydrogen bonds are shown as dashed lines. **(A)** Superimposition of the apo (purple) and complex (green) protein structures shows a shift of the C-terminal alpha helix region of the protein in response to ligand binding. ARD and UBD are ACP recognition domains and uridine binding domains respectively. **(B)** Compound **1** in the active site. The unbiased Fo – Fc map is contoured at 2.0 σ. The chain IDs (A/B) indicate different monomers constituting the active site.

To elucidate the molecular interactions between these ligands and LpxA/D, we solved the complex crystal structures of both LpxA and LpxD in complex with dual-binding compound **1**, and LpxA in complex with compound **2** (Figs 3 and 4). Interestingly, both compounds **1** and **2** have similar chemical characteristics, with two fused six-membered rings, and a 4–6 atom acyl chain with a terminal carboxyl group, a chemical species closely resembling its fatty acid substrate (Table 1).

The crystal structures of *P. aeruginosa* LpxA in its apo, substrate, and product-bound forms have previously been solved in our laboratory^24^. The LpxA crystal belongs to the P2_1_2_1_2_1_ space group with an asymmetric unit consisting of six monomers that form two separate biologically relevant homotrimers through a non-crystallographic 3-fold symmetry (Supplementary Table S1). This results in six active sites per asymmetric unit, which include 6 copies of the ligand; one at each of the dimer interfaces present in the homotrimers. For compound **1**, the complex structure with LpxA was solved at 2.10 Å resolution (Fig. 3). Two modes of binding conformations can be identified in terms of the relative location of the carboxyl group, with minor variations in the positioning of the carboxyl group seen within each bind mode (Fig. 3). In the first binding mode (exemplified by pose 1), the carboxyl group is within hydrogen bonding distance to the Gln157 main chain and His156 side chain (Fig. 3A). This type of conformation appears in 3 out of the 6 copies of the ligand present in the asymmetric unit. In the other binding mode (exemplified by pose 2), the ligand’s carboxyl group is positioned near the Gln157 side chain, forming a hydrogen bond (Fig. 3B). The rest of compound **1**, including the carbonyl group and the naphthalene ring, is placed identically in all poses. Notable intermolecular interactions include a hydrogen bond between the carbonyl oxygen and the Gly151 main chain. Additionally, the naphthalene ring forms multiple non-polar interactions with residues His118, Asn133, Val132, Ala136, Ala138, and Met169. Among these, Met169 is the so-called “hydrocarbon ruler” that ensures the length of the incoming acyl chain substrate is no longer than 10 carbon atoms^24^. Notably, when comparing the X-ray structure (pose 1) to the docking conformation of compound **1**, the poses are nearly identical with some minor differences in the exact position of the naphthalene ring and the carboxyl group (Supplementary Fig. S2). Comparing the apo and complex structures, there are minor side chain conformational changes in some monomers in response to ligand binding, including His156, Gln157, and Met169. Considering the structural variations among the six monomers of the asymmetric unit, the binding of compound **1** appears to select those conformations from the apo structure that can best accommodate specific ligand functional groups.

Aside from the acyl-chain binding pocket, compound **1** was found in a second binding pocket outside the active site at the dimer interface (Supplementary Fig. S3), where a second copy of the substrate UDP-GlcNAc was also identified in the previously published LpxA-substrate complex structure^24^. The naphthalene ring of compound **1** forms non-polar interactions with Leu3, Gly23 and Pro24, whereas the carboxyl group establishes favorable electrostatic interactions with Arg57. The functional relevance of this second substrate binding site is currently unclear. We hypothesize that the binding of compound **1** to this additional pocket may not directly impact the reaction catalyzed by LpxA, but it can complicate the interpretation of the SPR results, as observed in our experiments.

The complex structure of LpxA with compound **2** was solved at 2.00 Å (Fig. 3C,D). Corresponding ligand electron density is seen at all 6 active sites within the asymmetric unit. Similar to **1**, two binding modes were observed for compound **2**, with differences mainly in the conformation of the flexible side chain bearing the carboxyl group. Five out of the six copies of compound **2** adopt a binding conformation represented by pose 1, with the carboxyl group placed near His156 (Fig. 3C). In the other binding mode (pose 2), the carboxyl group is pushed away from His156 due to a new conformation of this residue. Many of the interactions formed by compound **1** with LpxA are also seen with compound **2**, especially for pose 1, including the hydrogen bonds between the carboxyl group and Gln157, and between the carbonyl group and Gly151. Meanwhile, the benzoxazine ring forms non-polar contacts with Ala136 and Val132, and the hydrocarbon ruler Met169. Whereas both the ring structures of compounds **1** and **2** are buried in the acyl chain pocket, the benzoxazine ring of compound **2** is pulled slightly outward, likely due to the hydrogen bonding interactions between the substituted amide group and residues His121 and Asp70 that have been proposed to be directly involved in catalysis^37,38^. The docking result predicted that compound **2** would be buried deeper in the acyl chain pocket, similar to **1** (Supplementary Fig. S2). This is likely due to the protein template used in the rigid docking procedure, which failed to account for the HB interactions with His121, Asp70 and Gln157.

Our LpxD complex structure with compound **1** (2.60 Å) showed distinguishable electron density corresponding to the ligand at the dimer interface formed via crystallographic symmetry (Fig. 4). The fitted binding pose suggests that there are two hydrogen bonds stabilizing the protein-ligand complex, both with the backbone nitrogen atoms of two glycine residues, Gly272 and Gly278, from the adjacent monomers forming the active site. Additionally, compound **1** establishes extensive non-polar interactions with residues Ala253, Gly257, Ala271, Leu276, Val277 and Met293, which may also be critical for the hydrophobic contacts with the substrate acyl chain. Indeed, structural alignments with the previously solved *Chlamydia trachomatis* LpxD structure (PDB 2IUA)^16^ reveal the decanoyl acyl chain of the substrate binds within the same pocket as the naphthalene ring of compound **1**.

Two conformational changes are observed in LpxD upon ligand binding, one being Ser259. In its apo form, the Ser259 side chain normally points inwards towards the active site. However, the naphthalene ring of compound **1** forces this residue to rotate outwards to avoid steric clash (Fig. 4B). An additional, unexpected shift of 3–5 Å is also seen outside of the active site, in the distal α-helical ACP recognition domain (ARD) domain (Fig. 4A), comparable to the changes observed between the apo structure of *E. coli* LpxD (PDB 3EH0)^16^ and the ACP bound forms (PDB 4IHF, 4IHG, and 4IHH)^39^. Considering the functional role of this domain in binding and releasing ACP bearing the acyl chain substrate, it is possible that this structural shift may suggest an intramolecular network responsible for coordinating ACP binding and enzymatic catalysis^39^.

## Discussion

Despite the important role of LpxA and LpxD in lipid A biosynthesis and their potential as antibiotic targets, small molecule inhibitor discovery has been lacking against these two enzymes. Many details of their enzymatic reactions also remain unclear, especially regarding how ACP binding influences the progression of the reaction, and vice versa. Our results not only provide the first examples of non-substrate small molecule ligands targeting LpxA and LpxD, but also demonstrate the possibility of designing dual-binding compounds active against both enzymes. In addition, our LpxD crystal structures shed light on previously uncharacterized structural features that may deepen our understanding of LpxD structure and function.

**Figure 5:**
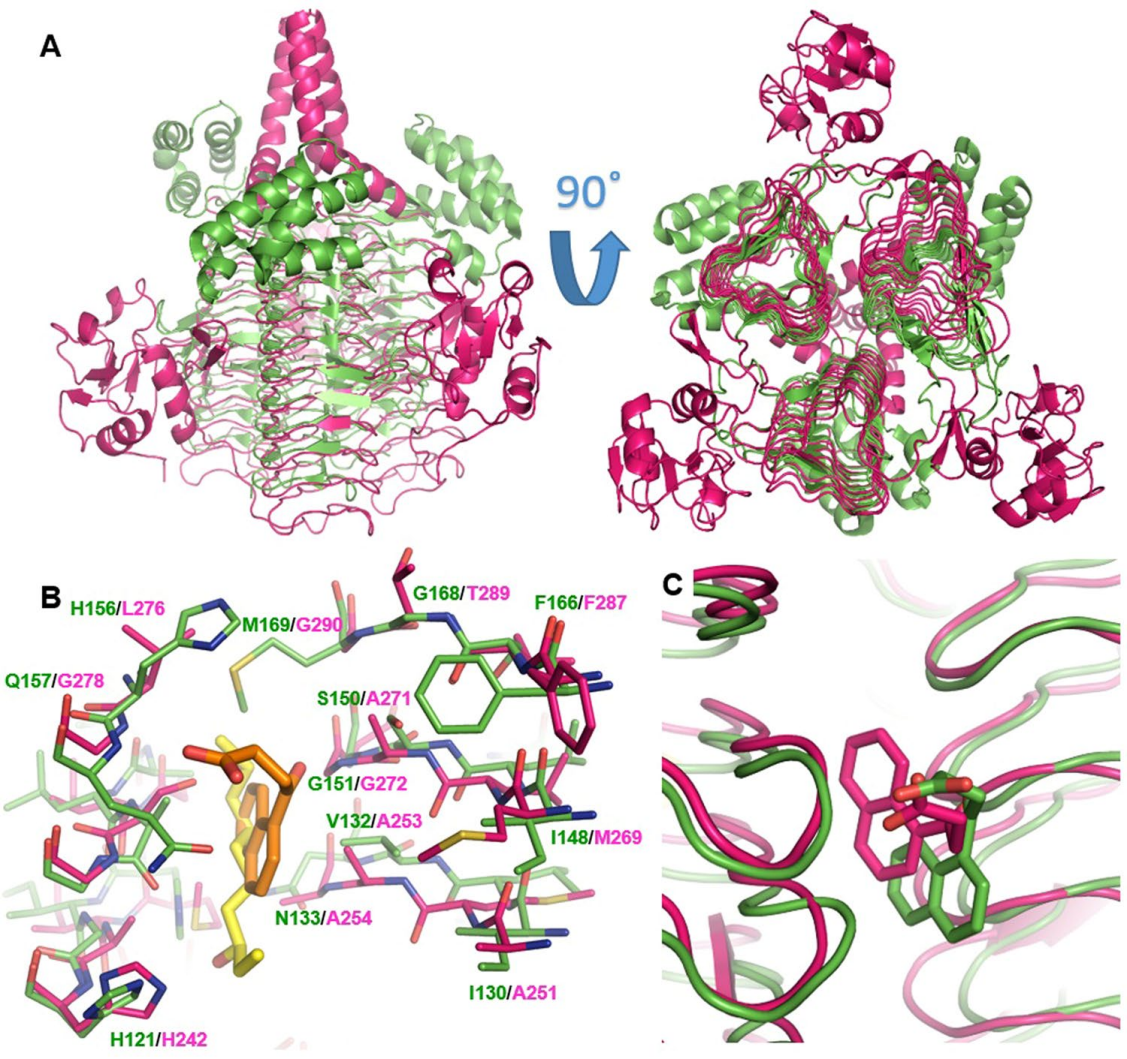
Structural comparison of *P. aeruginosa* LpxA and LpxD. (**A)** Superimposition of the overall LpxA (green, PDB ID: 5DG3) and LpxD (magenta) trimers shows high levels of conservation for the backbone structure. **(B)** Similarities in the binding hot spots of LpxA (green) and LpxD (magenta). The acyl-chain of the LpxA-product complex (yellow, PDB ID: 5DG3) and compound **1** (orange) from the new LpxA complex structure are shown to indicate the active site. His121 and His242 are the catalytic histidine in LpxA and LpxD respectively. **(C)** Compound **1** exhibits comparable binding poses in LpxA (green) and LpxD (magenta).

LpxA and LpxD are functionally and structurally similar, sharing a homotrimer architecture and conserved active site features, especially in the acyl-chain binding pockets that reside in the dimer interface of the β-helix trimer core (Fig. 5A). The similarities between the acyl-chain binding pockets of LpxA/D are highlighted by the dual-inhibitor peptide RJPXD33 for *E. coli* LpxA and LpxD (with *K*_*d*_ of 20 μM and 6 μM respectively)^27^, whose complex crystal structure with LpxA reveals potential hydrogen bond interactions with the protein backbone amide groups on the β strands, as well as non-polar interactions with protein backbone and side chains^28^. The same backbone functional groups, as well as similar side chain moieties, can be found in the acyl-chain binding pocket of LpxD. Our determination of the *P. aeruginosa* LpxA and LpxD crystal structures further demonstrates the conservation of binding hot spots between the two enzymes at the atomic level. Similar to the previously solved *E. coli* enzyme structures, the acyl-chain binding pockets in the *P. aeruginosa* LpxA and LpxD present the same backbone functional groups (Fig. 5B). Many side chains are also conserved between the two enzymes (e.g., Gly151/Gly272 from LpxA and LpxD respectively, and in the same order below), Phe166/Phe287, His121/His242 (the catalytic histidine), or present the same functional groups (e.g., Cβ atom of Ser150/Ala271, non-polar hydrocarbons of Val132/Ala253 and Ile148/Met269) (Fig. 5B).

One of the challenges of inhibitor discovery against LpxA and LpxD is the lack of an effective activity assay due to the nature and complexity of their reactions. Peptide inhibitor development has relied on biochemical experiments with radio-labeled substrates^29^, or competition assays with fluorescent peptides^27,40^, followed by structural analysis of validated hits. Our results have demonstrated the potential of combining SPR and X-ray crystallography as an alternative and efficient strategy for small molecule discovery. Despite the challenges posed by certain compounds, such as compound **1**, SPR provides an efficient method to detect direct binding to LpxA and LpxD, whereas X-ray crystallography offers a lower throughput but complementary approach to verify binding and to reveal binding modes for lead optimization. We hypothesize that the technical difficulty with compounds such as **1** in our SPR assay may have primarily originated from multiple binding modes and copies of ligand in the active site, considering the relatively small size of **1** and the large surface of the acyl-chain binding pocket especially in LpxD. Indeed, in the LpxA complex, compound **1** was found in a second pocket at the dimer interface outside the active site. Additionally, there was some residual weak electron density in the active site of LpxD complex crystal with compound **1** suggesting that another ligand copy could possibly be accommodated, although its low occupancy prevented it from being unambiguously identified and modeled at the current resolution. The complexity of ligand binding may be further amplified due to increased thermal motion at the experimental temperature of SPR (25 °C) in comparison to the cryogenic condition of X-ray diffraction data collection (~ −173 °C). Nonetheless the lowest-energy conformations of compounds **1** and **2** in our crystal structures provide valuable information about the protein binding hot spots and future compound modifications.

The dual-binding activity of several of our novel ligands has further demonstrated the feasibility of small molecule inhibitor discovery targeting LpxA and LpxD simultaneously. As shown by the complex structures of compound **1** bound to LpxA and LpxD, the ligand adopted similar binding poses in the two acyl-chain binding pockets, revealing both structural similarities and differences that can guide future dual inhibitor development (Figs 3 and 4B). The charged and polar functional groups of compound **1** interact with backbone amide groups shared by both LpxA and LpxD. The naphthalene ring is nestled in the hydrophobic pocket that recognizes the acyl-chain. Compared with the LpxA structure, the ligand’s aromatic ring goes deeper into the LpxD structure (Fig. 5C), consistent with a slightly larger acyl-chain binding pocket in LpxD.

The two complex structures also shed light on additional shared binding hot spots that can be exploited for future lead optimization. Particularly Phe166/Phe287, Val132/Ala253, and Ile148/Met269 (in LpxA/LpxD, respectively) provide large hydrophobic binding surfaces that can be very valuable for enhancing ligand binding affinity for both proteins (Fig. 5B). The binding affinities of our current small molecules are still relatively modest, with the *K*_*d*_ of the compounds ranging from 2.1 to 36.7 μM. In preliminary studies using laboratory *P. aeruginosa* strains, compound **2** and **5** did not show significant inhibition of bacterial growth at 256 µg/mL(data not shown). Interestingly, the *K*_*d*_ values of these new compounds are comparable to those of RJPXD33 for *E. coli* LpxA and LpxD^28^. Another previously developed *E. coli* LpxA inhibitor, peptide 920, and its recent truncation analog CR20, showed more potent activity against *E. coli* LpxA, with *K*_*i*_ values of around 50 nM^26,29^. Comparing the *E. coli* LpxA crystal structures of the peptide inhibitors illustrates more extensive interactions between LpxA and peptide 920/CR20 than RJPXD33. There are also more intramolecular interactions in peptide 920 and CR20, which adopt a hairpin conformation, in contrast to the extended configuration of RJPXD33. By leveraging additional binding surfaces in *P. aeruginosa* LpxA and LpxD, future development of our current small molecule scaffolds can lead to more active inhibitors that may also be cell permeable. In comparison, all the peptide inhibitors had to be expressed inside the cell in order to achieve their bactericidal effects.

The reaction catalyzed by LpxD follows a sequential ordered mechanism where acyl-ACP binds first, and holo-ACP dissociates last after the acyl chain is transferred to UDP-3-O-(R-3-OHC_10_)-GlcNAc substrate^41^. The recent determination of a series of *E. coli* ACP crystal structures in various complexes with LpxD provided important insights into the complex interactions between ACP and LpxD, and ligand conformations in the acyl-chain binding pocket crucial to ACP release^39^. Aside from a β-coil motif, the ARD consists mostly of the C-terminal α-helix (Fig. 4). One of the most interesting observations in the LpxD complex structure is how ligand binding in the acyl-chain binding pocket triggers movement in the distal C-terminal helix, suggesting possible cross-talk between the catalytic pocket and ACP binding site. A similar movement of the C-terminal helix is also observed in the *E. coli* LpxD complex structures with ACP, when comparing them to the apo structure. Our complex structure suggests the possibility of coordination between substrate binding in the active site and ARD binding by ACP. However, the potential functional implication of this observation remains to be investigated.

Our new LpxD crystal structures also shed light on a previously uncharacterized magnesium ion at the center of the trimer core. We hypothesize that a magnesium ion can potentially stabilize the trimer. Whereas the presence of magnesium was important for LpxD crystallization, our initial attempts to evaluate the influence of magnesium on LpxD stability in solution failed to produce conclusive results by varying magnesium or EDTA concentrations (data not shown). This is likely due to interactions of excessive amounts of magnesium or EDTA with other parts of the protein. Interestingly, in one of the *E. coli* LpxD structures (PDB ID: 4IHF), similar densities of a heavy atom with six coordinated waters were also observed at the exact location of the trimer core, but the heavy atom was modeled as Cl^-^ possibly because of the high concentration of Cl^-^ in the crystallization buffer^39^. However, it is highly likely that this density in *E. coli* LpxD also corresponds to a positively charged divalent ion, based on the water coordination pattern and the partially negatively charged protein environment due to the presence of a large number of backbone carbonyl groups. Together with our new data, the *E. coli* LpxD complex (PDB ID: 4IHF, 2.10 Å resolution) and the previously determined *P. aeruginosa* apo LpxD (PDB ID: 3PMO, 1.30 Å resolution) represent the highest resolution LpxD crystal structures, all of which appear to contain a structural divalent ion. It is possible that this divalent ion is present in the other LpxD crystal structures, but not visible in the electron density maps due to the diffraction resolution and data quality. Taken together, these observations suggest a possible structural role of the ion that merits further analysis.

## Conclusion

LpxA and LpxD are both promising targets for new antibiotic agents against GNB such as *P. aeruginosa*. Our studies have identified novel small-molecule scaffolds that can serve as starting points for future inhibitor discovery, including dual-binding compounds that take advantage of the structural and functional similarities shared by these two enzymes. Furthermore, the reported X-ray structures for LpxA and LpxD represent the first published structures of these enzymes complexed with non-substrate small molecule ligands. Though it was not the primary goal of this project, these complex structures also reveal interesting new structural features, most notably the α-helical shift in the ARD of LpxD following ligand binding. These observations offer new avenues for future investigation into the function of these proteins.

## Methods

### Materials

All reagents and chromatography supplies were purchased from Fisher Scientific. Crystal screens were purchased from QIAGEN. Compound **1** was purchased through Princeton BioMolecular Research, compounds **2**, **4**, and **5** were purchased through Enamine, and compound **3** was purchased from Vitas-M. The purity was >90% for all purchased compounds based on the information provided by the commercial sources. As control, LC-MS was used to verify the purity and molecular weight of the most active compound (compound **5**). The result confirmed the information provided by the vendor.

### Molecular docking

DOCK 3.5.54^42^ was used to dock the lead-like and fragment subsets of the ZINC^31^ database (~4.4 million compounds) into the previous solved product complex structure of *P. aeruginosa* LpxA (PDB ID: 5DEP)^24^. Multiple iterations of docking simulations were performed, each with minor alterations to conformational sampling space and side chain partial charges to favor conformations that would form hydrogen bonds with the targeted residues in the acyl chain binding pocket, such as Gln157, Gly151, and Met169. The final top 1000 ranking compounds were visually analyzed. The compounds that displayed the best complementarity and chemical diversity were selected and purchased for screening using X-ray crystallography and SPR.

### Construction and purification of recombinant LpxA and LpxD

LpxA was purified as previously described^24^. The N-terminal His-tagged *P. aeruginosa* LpxD was initially constructed the same as the published structure^20^. It was further altered by introducing deletions to the first two residues of the LpxD sequence using QuikChange Site-Directed Mutagenesis Kit (Stratagene) and gene specific oligonucleotide primers (forward primer [5′-GAA AAC TTG TAT TTC CAG GGC AGT ACC TTG TCC TAC ACC-3′] and reverse primer [3′- GGT GTA GGA CAA GGT ACT GCC CTG GAA ATA CAA GTT TTC-5′]). The pETMHL plasmid containing the N-terminal His-tagged *P. aeruginosa* LpxD with a 2 amino acid deletion sequence was transformed into cells, which were then grown in a 50 mL overnight culture of LB media with 35 µg/mL of chloramphenicol and 50 µg/mL of kanamycin at 20 °C. Then 10 mL of the overnight culture was added to 1 L of LB media containing 1 mL of 35 µg/mL of chloramphenicol and 50 µg/mL of kanamycin each. These cells were incubated at 37 °C for 4 hours until they reached an OD_600_ of 0.6 to 0.8. Then induction was carried out by adding 1 mL of 0.5 M IPTG, followed by further incubation at 20 °C overnight. The culture is then centrifuged at 5,000 *g* for 10 minutes and the pellet is resuspended in 10 mL of the lysis buffer (20 mM Tris-HCl pH 8.4, 300 mM NaCl, 10% glycerol v/v, and 20 mM imidazole). The cells were thawed on ice and transferred to a 40 mL beaker. The cells were then sonicated on a 10 second sonication/15 second rest cycle for a total of 15 minutes at an amplitude of 6. The lysate was centrifuged at 40,000 x g for 40 minutes at 4 °C. The supernatant was then filtered and loaded onto a HisTrap affinity column and eluted with a linear gradient spanning 10–500 mM imidazole. The fractions containing LpxD were collected, pooled, and concentrated down using an Amicon filter. The concentration of the protein was checked using UV_280_. The protein was loaded to a HiLoad 16/60 Superdex 75 column for additional purification along with the storage buffer (20 mM Tris-HCl pH 8.6 and 250 mM NaCl). The protein eluted at a peak consistent with the size of the trimeric form. The LpxD was stored at −80 °C at 35.8 mg/mL. The purity of the protein was determined by SDS-PAGE to be >95%.

### Construction and purification of biotinylated avidity(Avi) tagged LpxA & LpxD

The LpxA and LpxD genes were inserted into BamHI and HindIII site of pAvibir plasmid. The plasmids were transformed into Rosetta DE3 pLysS cells, which were then grown in an overnight culture of 50 mL LB media with 100 µg/mL ampicillin and 35 µg/mL chloramphenicol at 20 °C. Then 10 mL of the overnight culture was added to 1 L of LB media again with 100 µg/mL ampicillin and 35 µg/mL chloramphenicol and incubated at 37 °C until OD_600_ was at least 0.6 but less than 0.8. Protein expression was induced by adding 1 mL of 0.5 M IPTG, along with 10 mL of fresh biotin solution at a concentration of 5 mM. The culture was then incubated at 20 °C with vigorous shaking overnight. Cells were harvested by centrifuging the solution for 10 minutes at 4000 *g* at 4 °C. The supernatant was discarded and the cell pellet was then resuspended in 10 mL of the lysis buffer (10 mM Tris pH 8.4, 300 mM NaCl, 20 mM Imidazole, and 10% glycerol v/v) and a single dissolved protease inhibitor tablet. The resuspended cell pellet was then transferred to a 50 mL beaker for sonication. The cells were then sonicated on a 10 second sonication/15 second rest cycle for a total of 15 minutes at an amplitude of 6. The lysate was then centrifuged at 35,000 x g for 35 minutes at 4 °C. The supernatant was filtered and loaded onto a HisTrap affinity column and eluted with a linear gradient spanning 10–500 mM imidazole. The fractions containing protein were collected, pooled, and concentrated down using an Amicon filter. The concentration of the protein was checked using UV_280_. The protein was the loaded to a HiLoad 16/60 Superdex 75 column for additional purification along with the storage buffer (20 mM Tris pH 8.6, 250 mM NaCl, and 1 mM EDTA). The protein eluted at a peak consistent again with the trimeric form of the protein. The protein was stored at −80 °C at 7.9 mg/mL for LpxA and 16.1 mg/mL for LpxD. The purity of the protein was determined by SDS-PAGE to be >95%.

### LpxA and LpxD crystallization

LpxA was crystallized in 12% (w/v) PEG 1000, 0.2 M calcium acetate, and 0.1 M imidazole pH 7.0 with a drop ratio of 1 μl of protein to 2 μl of well solution and 0.5 μl of seed stock, as previously described^24^. LpxD crystals with a cuboidal-like morphology were grown in 12% (w/v) PEG 3350 and 0.2 M magnesium acetate with a drop ratio of 2 μl of protein to 3 μl of well solution and 0.5 μl of seed stock. The crystals appeared within 21–28 days and measured up to 0.1 mm in length. Complex crystals were obtained by transferring apo crystals into crystallization solution containing 20 mM of each respective compound and 10% DMSO, and soaked for 24 hours (LpxA) or 51 hours (LpxD). The crystals were then cryoprotected with crystallization buffer containing 25% glycerol and immediately flash frozen in liquid nitrogen.

### X-ray diffraction data collection & processing

X-ray diffraction data were collected at the SER-CAT and SBC beamlines at the Advanced Photon Source (APS) within Argonne National Laboratory (ANL). All data sets were indexed and integrated using iMosflm and scaled with Scala in the CCP4 suite^43^. The LpxD structures were solved by molecular replacement using Molrep (CCP4) and the previously determined *P. aeruginosa* LpxD structure (PDB ID: 3PMO) as the model. All LpxA crystals structures were solved using the previously solved *P. aeruginosa* LpxA structure (PDB ID: 5DEM) as the starting model. All model rebuilding of the solved structures was done using Coot, while all the refinements were carried out in the program REFMAC5 also found in the CCP4 suite. All figures of protein structures were created with the use of PyMOL (Schrödinger).

### Surface plasmon resonance

All experiments were conducted using a Biacore 4000 instrument with a CM5 chip at 25 °C. Avi-LpxA (31.6 kDa, 7.9 mg/mL stock concentration) and Avi-LpxD (39.8 kDa, 16.1 mg/mL stock concentration) were used as ligands to capture onto the NeutrAvidin immobilized CM5 chip surface. NeutrAvidin (60 kDa, 10 mg/mL) was diluted in 10 mM sodium acetate buffer at pH 4.5 (1:50 dilution, 200 µg/mL diluted concentration) and immobilized to a level of ~18000 RU, using standard amine coupling chemistry. This NeutrAvidin was immobilized onto all spots (Ss) of all flow cells (FCs). PBS-P (20 mM Phosphate buffer, pH 7.4, 2.7 mM KCl, 137 mM NaCl, 0.05% v/v surfactant P20) was used as the immobilization running buffer. Flow cell 3 off all flow cells were used as the reference spot. Avi-LpxA was diluted (1:75 dilution, 105.3 µg/mL diluted concentration) in PBS-P and injected onto S1 of all FCs (FCs 1–4). Avi-LpxD was diluted (1:75 dilution, 214.7 µg/mL diluted concentration) in PBS-P and injected onto S5 of all FCs. The ligands were captured in the presence of PBS-P. Based on the captured response values, theoretical R_max_ values were calculated for the lowest and highest MW analytes. The R_max_ values assume 1:1 interaction mechanism. Overnight kinetics were performed for all compounds in the presence of PBS-P+ 1% DMSO buffer. Contact time and dissociation time used in screening experiments were 60 seconds and 300 seconds respectively. Injected analyte concentrations were 0 µM, 2.5 µM, 5 µM, 10 µM, 20 µM, and 40 µM. The compounds were injected in triplicate for each concentration. Data from overnight kinetics were evaluated by steady state affinity or 1:1 kinetics model fitting. The SPR response curves and fitted curves can be found in Supplementary Fig. S1.

### β-lactamase inhibition assays

CTX-M-14 and KPC-2 were purified as previously described^35,36,44^. The hydrolytic activity of CTX-M-14 and KPC-2 was determined using the β-lactamase substrate nitrocefin in a PBS-P+ buffer containing 200 mM phosphate buffer pH 7.4, 27 mM potassium chloride and 1.37 M sodium chloride, 0.5% (v/v) surfactant P20 supplemented with 2% DMSO. Nitrocefin hydrolysis was monitored using a BioTek Synergy Mx monochromator-based multimode microplate reader at 486 nm wavelength. For CTX-M-14 and KPC-2 inhibition assays, the nitrocefin concentration used was 20 µM and 10 µM, respectively. The Km of nitrocefin for CTX-M-14 is 22 µM and for KPC-2 is 10 µM. Compounds were used for IC50 measurements up to 3 mM based on their solubility in DMSO. The final protein concentration used in the reaction for CTX-M-14 and KPC-2 was 0.3 nM and 1 nM respectively. The protein was added last to initiate the reaction.

## Supplementary information

supplementary informations

## Data Availability

The atomic coordinates and structure factors for all LpxA and LpxD structures have been deposited in the Protein Data Bank (http://www.rcbs.org) under the accession numbers 6UEC, 6UED, 6UEE, and 6UEG.
