# Supplementary material for: Discovery of dual-activity small-molecule ligands of Pseudomonas aeruginosa LpxA and LpxD using SPR and X-ray crystallography
Source: Sci Rep. 2019 Oct 29;9:15450. doi: 10.1038/s41598-019-51844-z (PMC6820557; doi:10.1038/s41598-019-51844-z)
Supplement: Supplementary file 1 — supplementary informations [file 41598_2019_51844_MOESM1_ESM.pdf]

Supporting Information

Discovery of dual-activity small-molecule ligands of *Pseudomonas aeruginosa* LpxA and LpxD using SPR and X-ray crystallography

Kyle G. Kroeck†¤, Michael D. Sacco†¤, Emmanuel W. Smith†¤, Xiujun Zhang†, Daniel Shoun†, Afroza Akhtar†, Sophie E. Darch†, Frederick Cohen‡, Logan D. Andrews‡, John E. Knox‡, and Yu Chen†*

† Department of Molecular Medicine, University of South Florida, 12901 Bruce B. Downs Boulevard, Tampa, Florida 33612, United States

‡ Former employees of ACHAOGEN Inc., 1 Tower Place, Suite 400, South San Francisco, California 94080, United States

¤ These authors contributed equally to this work.

*Corresponding author: ychen1@health.usf.edu

Contents

Supplementary Table S1 .... S3

Supplementary Figure S1 .... S4

Supplementary Figure S2 .... S9

Supplementary Figure S3 .... S10

Supplementary Table S1. X-ray data collection and refinement statistics

Data Collection

	LpxD Apo	LpxD+**1**	LpxA+**1**	LpxA+**2**
Structure (PDB ID)	(6UED)	(6UEC)	(6UEE)	(6UEG)
Space Group	H3	H3	$P2_12_12_1$	$P2_12_12_1$
Cell Dimensions				
a, *b*, *c* (Å)	104.78	104.76	80.31	80.16
	104.78	104.76	82.56	82.52
	94.12	95.97	221.89	223.45
a, b, γ (°)	90	90	90	90
	90	90	90	90
	120	120	90	90
Resolution (Å)	50.00-1.55	32.96-2.60	77.33-2.10	55.43-2.00
No. Reflections	55962 (2461)	12038 (1220)	71637 (10722)	79561 (8270)
R_{merge} (%)	5.8 (50.7)	10.5 (53.2)	9.4 (49.8)	9.2 (45.4)
<*I*> / *ó*<*I*>	10.6 (2.0)	11.9 (2.7)	10.2 (2.8)	13.2 (1.7)
Completeness (%)	96.23 (98.1)	99.6 (99.8)	82.6 (85.6)	87.6 (76.0)
Redundancy	11.4 (11.0)	2.6 (2.7)	4.0 (3.9)	5.1 (5.3)

Refinement

Resolution (Å)	32.71-1.55	32.99-2.60	77.33-2.10	55.43-2.00
R_{work}/R_{free} (%)	17.57/19.26	20.89/28.85	21.83/25.00	17.62/23.63
**No. Heavy Atoms**				
Protein	2606	2477	11790	11790
Ligand/Ion	1	18	108	137
Water	362	58	262	216
***B*-Factors (Å^2)**				
Protein	19.99	59.52	28.97	26.54
Ligand/Ion	26.6	36.66	29.31	21.55
Water	29.45	24.34	18.92	16.66
**Ramanchandran Plot**				
Most Favored Region(%)	98.8	89.9	95.2	95.0
Additionally Allowed (%)	1.2	8.0	4.6	4.6
Generously Allowed (%)	0.0	2.1	0.2	0.4

* Data were collected from a single crystal for each structure. Values in parentheses represent highest resolution shells.

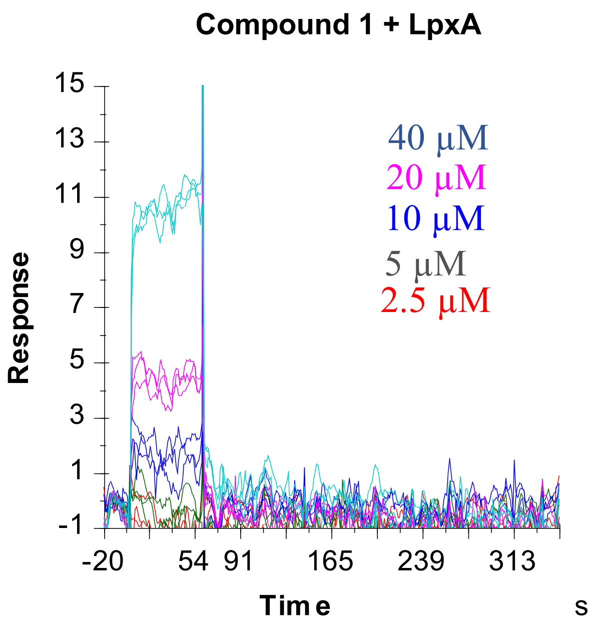
Compound 1 + LpxA
Response
15
13
11
9
7
5
3
1
-1
-20
54
91
165
239
313
Time
s
40 μM
20 μM
10 μM
5 μM
2.5 μM

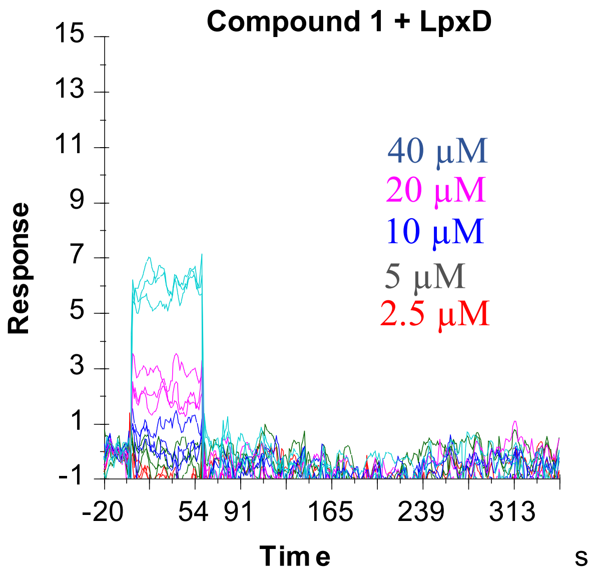
Compound 1 + LpxD
Response
15
13
11
9
7
5
3
1
-1
-20
54
91
165
239
313
Time
s
40 μM
20 μM
10 μM
5 μM
2.5 μM

Compound 2 + LpxA

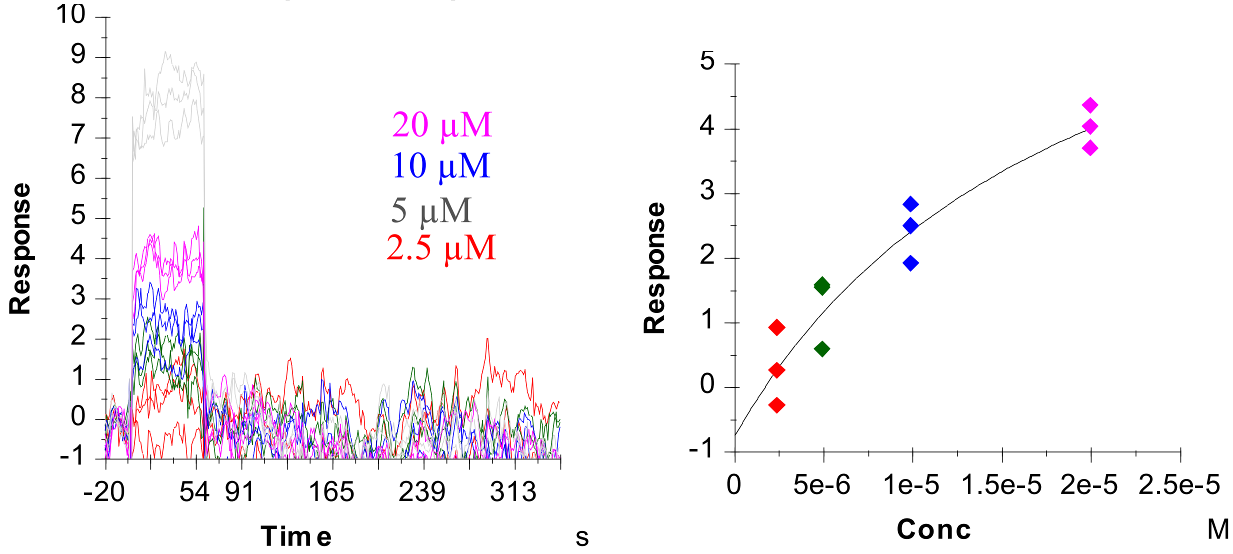

Compound 2 + LpxD

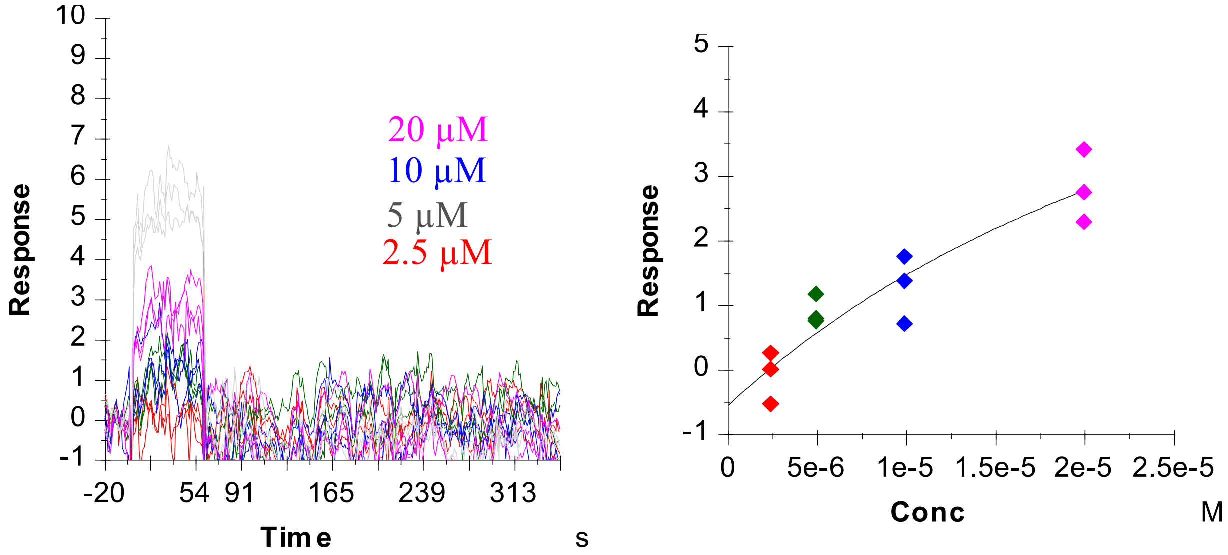

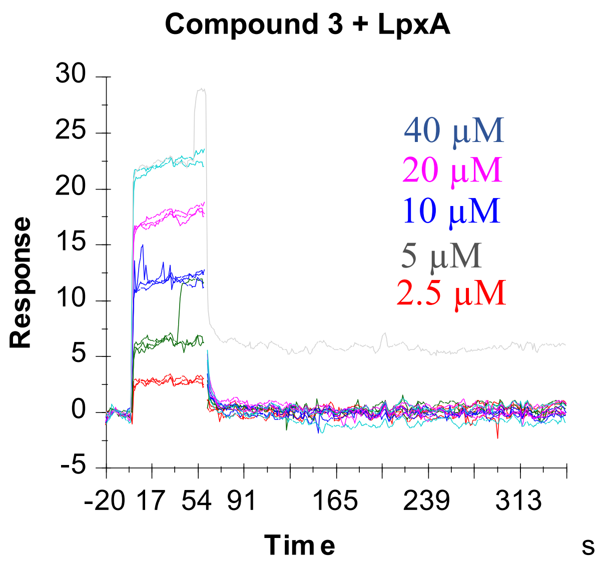
Compound 3 + LpxA
40 µM
20 µM
10 µM
5 µM
2.5 µM
Response
Time
s
30
25
20
15
10
5
0
-5
-20
17
54
91
165
239
313

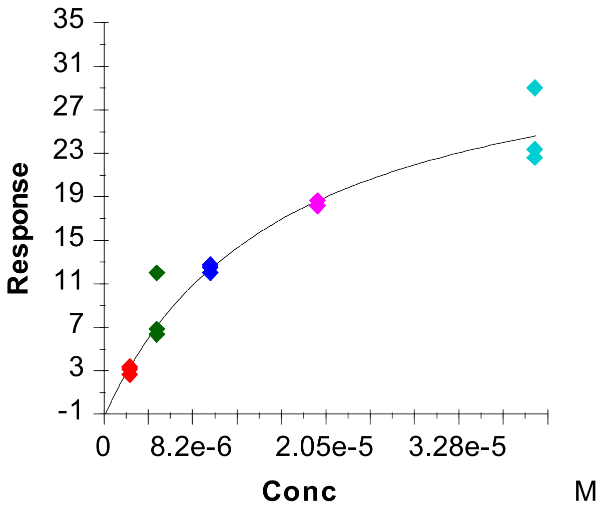
Response
Conc
M
35
31
27
23
19
15
11
7
3
-1
0
8.2e-6
2.05e-5
3.28e-5

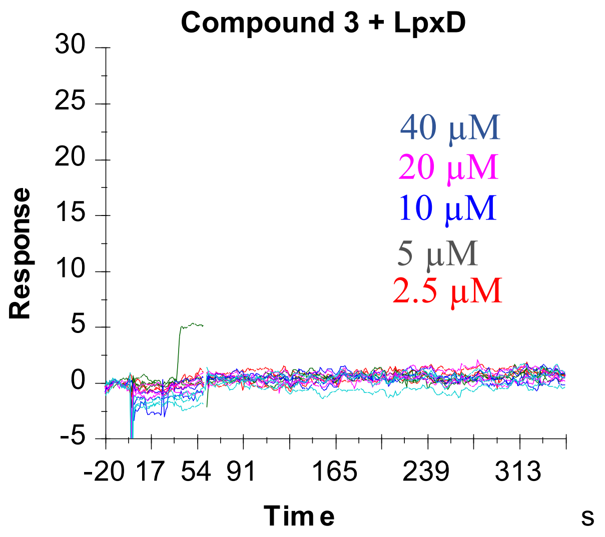
Compound 3 + LpxD
40 µM
20 µM
10 µM
5 µM
2.5 µM
Response
Time
s
30
25
20
15
10
5
0
-5
-20
17
54
91
165
239
313

Compound 4 + LpxA

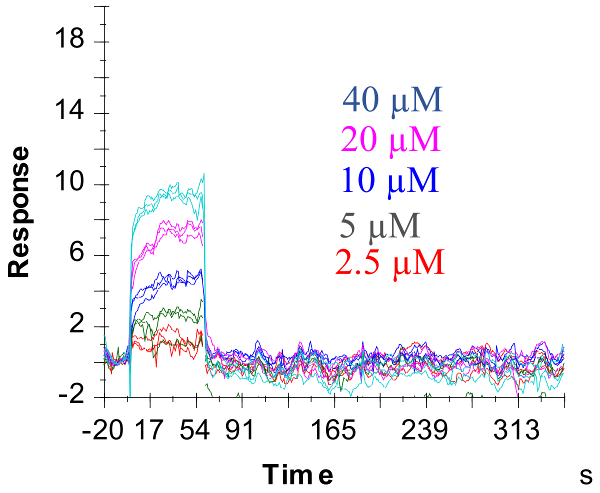

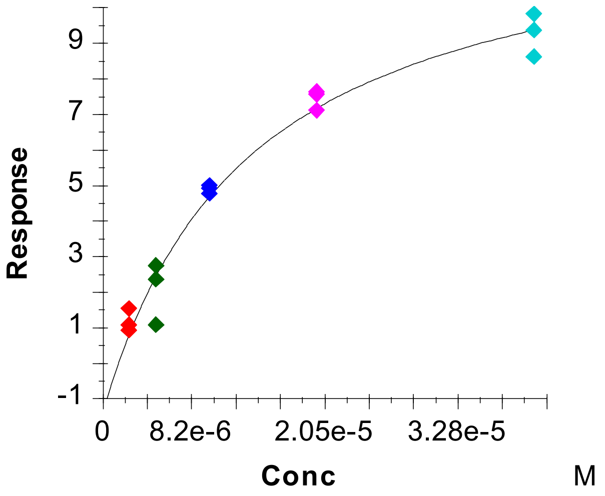

Compound 4 + LpxD

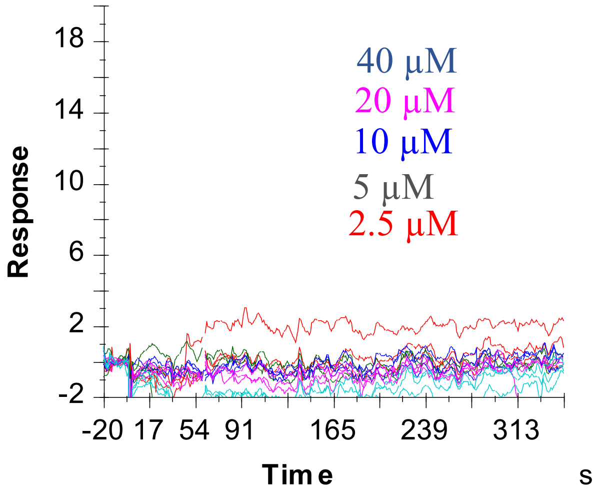

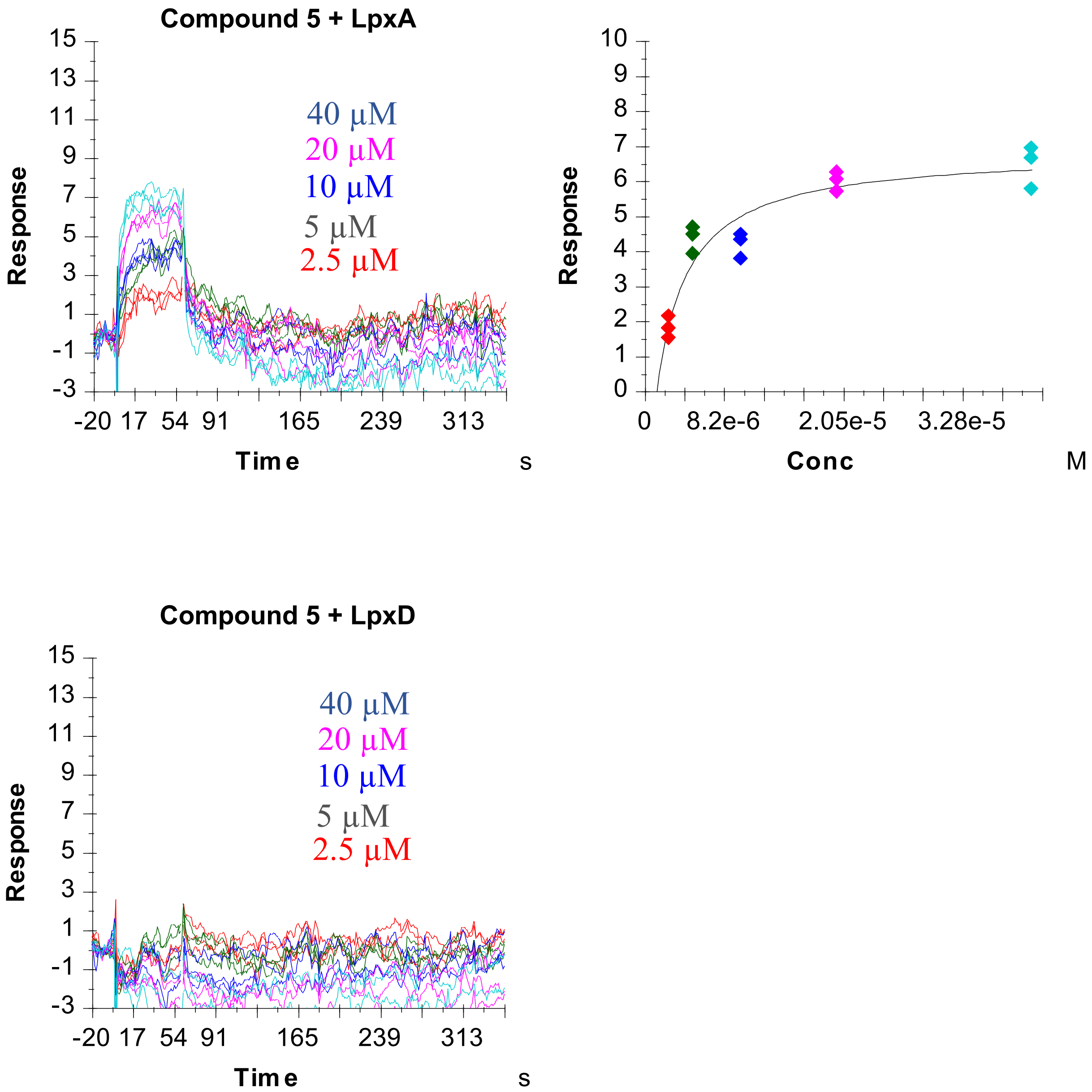

Supplementary Figure S1. Surface Plasmon Resonance Response Curves. The SPR sensorgrams for each compound (done in triplicates for each concentration) are shown on the left, and the fitted dose-response curve on the right for true binders. Sensorgrams in grey color are excluded in evaluation due to poor fitting.

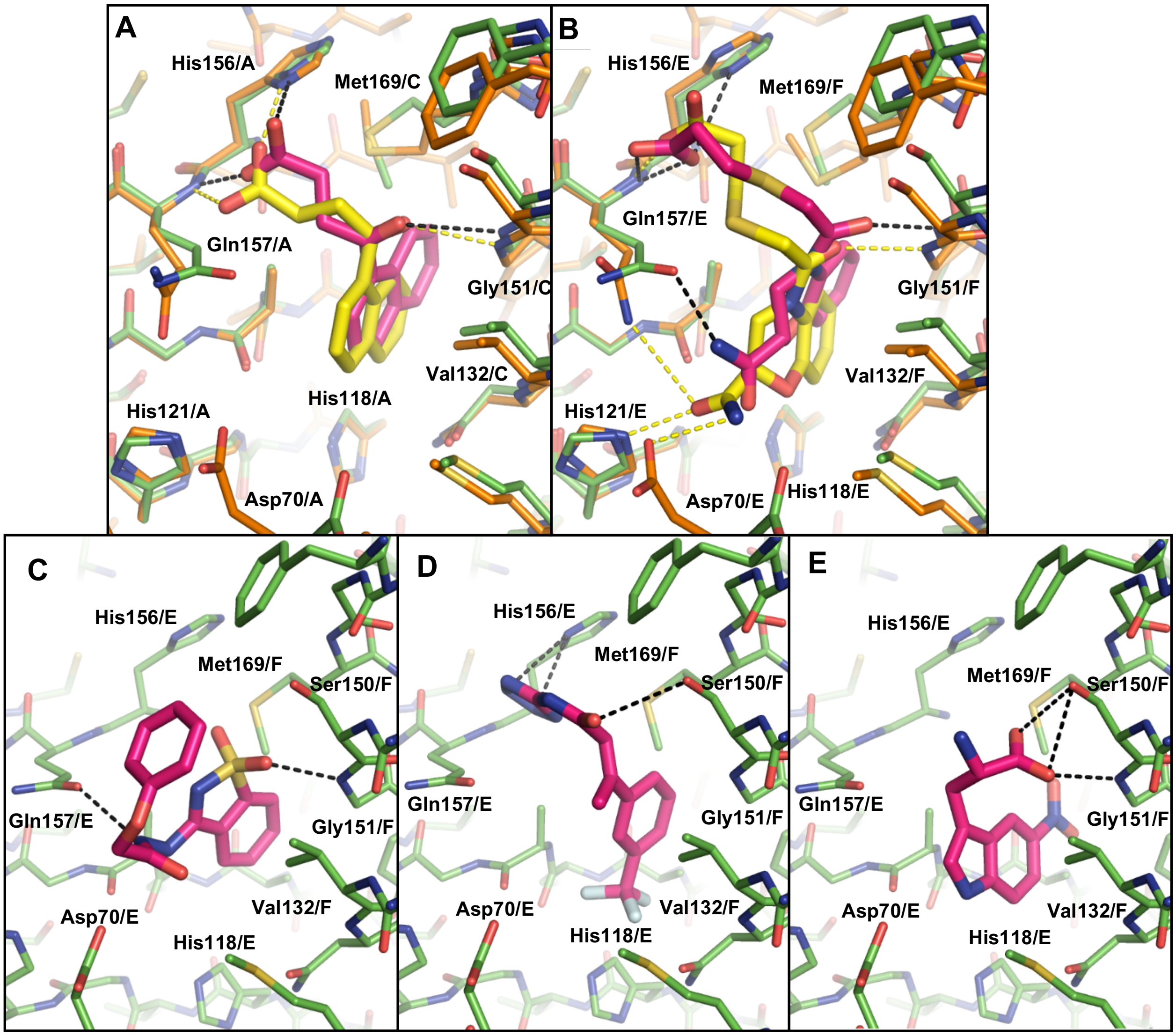

Supplementary Figure S2. Docked Poses of LpxA Ligands. For the docking results, the protein template and ligand are colored in green and magenta respectively, with potential hydrogen bonds shown as black dashed lines. For complex crystal structures of compounds **1** and **2**, the protein and ligand are colored in orange and yellow respectively, with potential hydrogen bonds shown as yellow dashed lines. **(A)** Superimposition of X-ray complex structure and the docked structure for compound **1**. **(B)** Superimposition of X-ray complex structure and the docked structure for compound **2**. **(C)** Predicted docked pose for compound **3**. **(D)** Predicted docked pose for compound **4**. **(E)** Predicted docked pose for compound **5**.

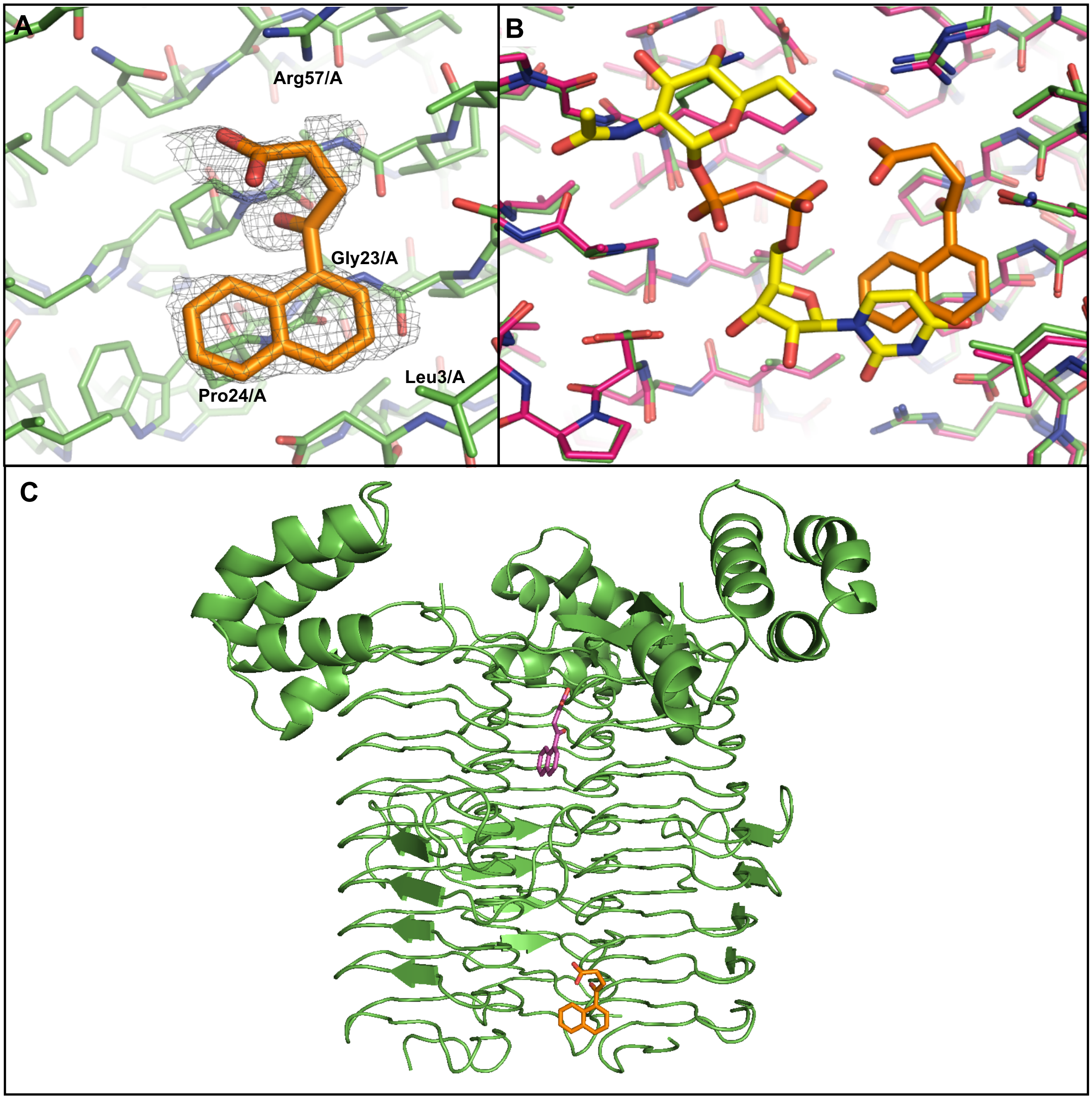

Supplementary Figure S3. Compound 1 Found Present in Secondary Binding Site on LpxA. Compound **1** found at the secondary binding site is colored in orange and the protein is displayed in green. **(A)** The unbiased Fo – Fc map contoured at 2.0 σ for compound **1**. **(B)** Structural alignment of LpxA complex structure with compound **1** and the previously determined LpxA complex structure with UDP-GlcNAc (PDB ID:5DEP) at the same secondary binding site. For the substrate complex, UDP-GlcNAc and the protein are colored in yellow and magenta respectively. **(C)** Relative positions of the two binding sites found on LpxA. The copy of compound **1** found in the acyl chain binding pocket is colored in purple.
